# Apparent total tract digestibility and palatability of extruded diets with graded levels of whole soybeans by dogs

**DOI:** 10.3389/fvets.2023.1137788

**Published:** 2023-05-18

**Authors:** Hee S. Kim, Sang Li, Yi Zheng, Charles G. Aldrich

**Affiliations:** Grain Science and Industry Department, Kansas State University, Manhattan, KS, United States

**Keywords:** animal nutrition, companion animals, dog, legumes, nutrient digestibility, stool quality, pet food

## Abstract

Fat has high energy density and is considered one of the primary energy sources for dogs, however, increasing fat level in dry dog food has been challenging due to the lubrication and limitation of the coating system. The objective was to determine the effect of whole soybeans (WSB) on nutrient digestibility, stool quality, and palatability by dogs. The corn gluten meal, chicken fat, and brewers rice were replaced by WSB at 10, 20, and 30% (WSB10, WSB20, and WSB30, respectively) in the base diet (WSB0). Twelve beagles were randomly assigned. The digestibility trial was duplicated 4 × 4 Latin square design where dogs were allowed a 9-d adaptation followed by a 5-d total fecal collection for each period. Least-square means were analyzed with a single degree of freedom contrasts and significance at α = 0.05. Palatability was determined with a 2-bowl test by 20 beagles for 2 d with each WSB diet compared to the WSB0. First choice preference between two diets and total food consumption were recorded. Individual intake ratios (IR) were calculated (intake of each diet/total intake) for each dog. First choice (FC) was analyzed by a Chi-square probability, and the diet consumption was compared by a Wilcoxon signed rank test and a 2-way analysis of variance. Fecal moisture, output, and defecation frequency increased linearly (*P* < 0.05) as WSB increased. Apparent total tract digestibility of dry matter, organic matter, crude protein, fat, and gross energy decreased linearly (*P* < 0.05) as dogs fed the increased level of WSB. The fresh fecal pH in dogs decreased linearly (*P* < 0.05) as WSB content increased. The acetate, propionate, and the total short-chain fatty acid concentration increased linearly (*P* < 0.05) while the total branched-chain fatty acid concentration decreased linearly (*P* < 0.05) as WSB increased. Dogs had greater (*P* < 0.05) FC for WSB diets than WSB0, but there was no difference among treatments for diet consumption and IR. In conclusion, additional thermal processing before extrusion may improve nutrient digestibility of WSB. The stool quality and palatability were not affected, and fermentation in hindgut increased by WSB by dogs.

## 1. Introduction

The U.S. pet industry had $50 billion in pet food and treats expenditures in 2021 (1). As the pet food market has grown, the categories have become more differentiated to meet the specific needs of animals and their owners. For example, working dogs have higher energy requirements compared to dogs at maintenance (2). Fat has high energy density and is considered one of the primary energy sources for dogs, and thus, fat level needs to be adjusted to maintain appropriate caloric intake as activity level increases (3). Glucose oxidation is the principal source of energy of energy expenditure in dogs, but fat oxidation still provides some energy and may affect maximal energy expenditure in dogs undertaking endurance exercise (4). Interestingly, feeding diets high in polyunsaturated fats may improve olfactory ability (5) suggesting that an increase in fat level from vegetable oils may be even more beneficial for some working dogs. However, increasing fat level in dry dog food has been challenging due to the lubrication in the extrusion process and limitation in the coating system (6). Kim et al. (7) reported that introducing intrinsic fats derived from whole soybeans (WSB) had less negative impact on processing compared to a liquid fat.

The use of vegetable proteins in animal foods has become important to address consumer concerns about the health and safety of animal protein byproducts (8). Soybean is the most essential oilseed crop grown in the U.S., and soybean meal (SBM) is the major source of protein for the livestock feed. In a previous report, the WSB contained 38.50% (dry weight basis) crude protein (6) and anti-nutritional factors that decreased the bioavailability in dogs. These results can be improved with proper heating to eliminate the anti-nutritional factors (9, 10). The WSB also consisted of ~8% soy hulls (11), and the soy hulls contained 63.8–81.2% of total dietary fiber [TDF; (12)]. Extrusion degraded the lignocellulosic structure and improved enzymatic hydrolysis of soybean hulls (13). Thus, extrusion processing may improve the bioavailability of fibers in soybean hulls in monogastric animals (14). Further, the soluble fiber and oligosaccharides (OS) in soy may be beneficial for dogs serving as a prebiotic fiber which can be fermented and utilized as an energy source by the hindgut microbiome (15). Soy OS refer to galactosyl-sucrose raffinose and stachyose in soybeans that are non-digestible (16). Hernot et al. (17) reported that the galactooligosaccharides caused the greatest production of total SCFA at all time points during *in vitro* fermentation experiments in human subjects, compared to fructans and polydextrose.

There have been numerous research reports that described an increase in bioavailability of the extruded soy-based products fed to rainbow trout, broiler chicks, and pigs (18–20). What has not been elucidated is an optimal level of whole soybean OS for dogs. Our hypothesis was that the WSB inclusion in extruded diets would not have a negative impact on nutrient digestibility and would benefit the gut health of dogs. Therefore, the objective of this study was to determine the effects of incremental levels of WSB on the total tract apparent digestibility, stool quality, and palatability of extruded diets by dogs.

## 2. Materials and methods

All animal procedures were approved by the Kansas State University Institutional Animal Care and Use Committee (IACUC) under protocol #4097.

### 2.1. Experimental diets

Four diets were formulated to be nutritionally balanced for adult dogs (21). The corn gluten meal, chicken fat, and brewers rice were replaced by WSB at 10, 20, and 30% (WSB10, WSB20, and WSB30, respectively) in the base diet (WSB0, control; Table 1) in order to maintain the diets protein and calorie content. The experimental diets were formulated to be consistent with a premium dog food with high-protein and moderate level of fat (>25% CP and >12% CF). Diets were formulated to have similar nutritional composition and included titanium dioxide (TiO_2_; 0.4%) as an indigestible marker for determination of apparent total tract digestibility (ATTD) of dietary nutrients. As predicted by the formulation, the concentrations of minerals; calcium, phosphorous, potassium, magnesium, sodium, sulfur, manganese, copper iron, and zinc were similar among diets and met AAFCO (21) nutrient profile recommendations for adult dogs at maintenance.

**Table 1:** Diet formulas with analyzed nutrient compositions of the experimental diets with increasing levels of WSB (WSB0, 0%; WSB10, 10%; WSB20, 20%; and WSB30, 30%).

**Ingredient, %**	**WSB0**	**WSB10**	**WSB20**	**WSB30**
WSB	0.00	10.00	20.00	30.00
Corn	22.50	22.50	22.50	22.50
Wheat	22.50	22.50	22.50	22.50
Corn gluten meal, 60%	15.74	9.54	3.55	0.00
Rice, brewers	8.58	6.67	4.54	0.00
Chicken meal	15.00	15.00	15.00	15.00
Beet pulp	4.00	4.00	4.00	4.00
Salt	0.50	0.50	0.50	0.50
Dicalcium phosphate	0.45	0.45	0.45	0.45
Titanium dioxide	0.40	0.40	0.40	0.40
Potassium chloride	0.25	0.25	0.25	0.25
Choline chloride, 60% dry	0.20	0.20	0.20	0.20
Fish oil	0.20	0.20	0.20	0.20
Calcium carbonate	0.15	0.15	0.15	0.15
Vitamin premix^a^	0.15	0.15	0.15	0.15
Flaxseed	0.13	0.13	0.13	0.13
Trace Mineral Premix^b^	0.10	0.10	0.10	0.10
L-Threonine 98%	0.10	0.10	0.10	0.10
Dry natural antioxidant^c^	0.04	0.04	0.04	0.04
Chicken fat (topical)	8.02	6.13	4.25	2.34
Digest—dry dog flavor (topical)	1.00	1.00	1.00	1.00
**Analyzed nutrient composition**				
Moisture, %	5.30	7.70	8.12	8.47
- Dry matter basis -				
Ash, %	5.64	5.91	6.42	7.02
Crude protein, %	30.48	29.79	28.92	29.98
Acid hydrolyzed ether extract, %	11.66	12.75	13.20	13.72
Total starch, %	41.38	39.82	35.98	30.83
Total dietary fiber, %	10.19	11.99	12.97	16.16
Gross energy, kcal/kg	5,076.10	5,001.77	4,947.74	4,900.92
^*^Metabolizable energy, kcal/kg	3,506.20	3,520.10	3,393.50	3,294.55

a Vitamin premix: 5.51% moisture, 4.02% crude protein, 34.5% ash, 13.4% calcium, 17,162,999 IU/kg Vitamin A, 920,000 IU/kg Vitamin D, 79,887 IU/kg Vitamin E, 14,252 mg/kg thiamine, 4,719 mg/kg riboflavin, 12,186 mg/kg pantothenic acid, 64,736 mg/kg Niacin, 5,537 mg/kg pyridoxine, 720 mg/kg Folic acid, 70 mg/kg biotin, 22 mg/kg vitamin B12.

b Trace mineral premix: 0.66% moisture, 21.5% calcium, 0.02% sodium, 0.57% magnesium, 38,910 mg/kg iron, 11,234 mg/kg copper, 5,842 mg/kg manganese, 88,000 mg/kg zinc, 1,584 mg/kg iodine, 310 mg/kg selenium, 19% carbohydrate, and 1% crude fat.

c Dry natural antioxidant: mixed tocopherols, citric acid, rosemary extract, and soybean oil.

^*^Calculated value: metabolizable energy = (3.5 ^*^ crude protein, %) + (8.5 ^*^ acid hydrolyzed ether extract, %) + (3.5 ^*^ total starch, %).

Raw materials were blended by a commercial mill (Fairview Mills, Seneca, KS, USA) and were ground via a hammer mill to pass through a 2-mm screen. Whole soybeans were supplied by MKC (Manhattan, KS, USA). They were cleaned using a grain cleaner and ground with a hammer mill (model 18-7-300; Schutte Buffalo, NY, USA) to pass through a 1.19-mm screen (3/64”) at the Hal Ross Flour mill (Manhattan, KS, USA). Mixing of the dry raw materials and extrusion were conducted at a local extrusion pilot plant (ExtruTech Inc.; Sabetha, KS, USA) under procedures described previously by Kim and Aldrich (6). All ingredients except the chicken fat and digest (dry dog flavor) were mixed in a ration prior to single screw extrusion. Extrudates were dried post-extrusion and applied for chicken fat and dry digest coating. While the amount of chicken fat declined with each increment of added soybeans according to the study design, the topical addition of fat was maintained > 2.0% as is typical for the minimum acceptable level for commercial pet food production.

### 2.2. Animal feeding

Dogs were housed at the Large Animal Research Center at Kansas State University (Manhattan, KS, USA). Twelve healthy adult beagles (eight neutered male and four spayed female) of similar age (6.25 ± 0.45 years) and initial body weight (BW, 12.28 ± 1.51 kg) were individually housed in metabolic pens (1.83 m × 1.20 m) equipped with an acrylic-mesh floor to allow for the separation of urine and feces. All dogs selected for this study had a body condition score ranging between 5 and 6 on a 9-point scale, with 1 being very thin, 4 to 5 being ideal, and 9 being excessively obese (22). The dogs were maintained as six dogs per room in a temperature-controlled (23°C) modular building with automatic light timers set to 16:8 h (light:dark) for each 24-h cycle.

The dogs were randomly assigned and fed a specific diet (WSB0: corn-wheat based diet with no WSB; WSB10: diet with 10% WSB; WSB20: diet with 20% WSB; WSB30: diet with 30% WSB). Initial dietary intake on day 0 was determined by weighing the dogs and calculating the dogs' daily metabolizable energy (ME) requirements for an average for laboratory kennel dogs [130 ^*^ BW∧0.75; (23)]. The ME of the experimental diets was calculated using the predicted equations in dog foods [(8.5 ^*^ Crude fat) + (3.5 ^*^ Crude protein) + (3.5 ^*^ Nitrogen-free extract (NFE); (23))]. The food intake was calculated using the dogs' daily ME relative to the predicted ME for the diets. Food allowance was controlled for each animal and feeding twice daily (at 800 and 1,700 h) in equal portions at each meal. Orts were removed and weighed after the feeding. Throughout the study, dogs were weighed weekly, and their food allowance was adjusted by 5 or 10% for the subsequent week to maintain their BW. Water was provided for *ad libitum* consumption.

### 2.3. Sample collection

The study was conducted in a replicated 4 × 4 Latin square design consisting of four periods with 9 d of adaptation to the diet followed by 5 d of total fecal collection for a total duration of 56 d. Random assignment of experimental treatments to each of the 12 dogs was carried out with the aid of a Balanced Latin Square Design Excel spreadsheet-based program (24). After the 9 d of adaptation, fecal samples were collected and scored on a 5-point scale following the method used in Acuff and Aldrich (25) wherein: 1 = liquid diarrhea, 2 = very soft consistency, unformed stool; 3 = soft stool that retains shape; 4 = well-formed firm stool that does not leave residue when picked up; and 5 = very hard, dry stool. A fecal score range of 3.5–4 was considered ideal. The defecation frequency was determined by counting the number of feces excretions per dog per day. After scoring, feces were collected in individual whirl-pak bags, weighed, and stored frozen at −20°C until further analysis. During the 5-d collection period, one fresh fecal sample from each dog was immediately collected (within 15 min of excretion) and measured for pH by inserting a calibrated glass-electrode pH probe robe (FC240B, Hanna Instruments, Smithfield, RI) directly into the sample in triplicate. After measuring the pH, the fresh fecal samples were collected in three 2-mL microcentrifuge tubes and stored at −80°C for further analysis of SCFA, BCFA (branched-chain fatty acids), and ammonia. After each collection period, bagged fecal samples were thawed at room temperature, pooled by dog, weighed, and dried in a forced air oven at 55°C for up to 48 h until the moisture level was below 10%. This initial drying step avoided bacterial or mold growth until fecal nutrients were analyzed. The partially dried fecal samples were also weighed, and the values were used when calculating the DM (dry matter) of the fecal samples. Diet samples and partially dried fecal samples were ground using a laboratory fixed blade impact mill (Retsch, type ZM200; Haan, Germany) fitted with a 1-mm screen and stored in lidded glass jars at room temperature in preparation for chemical analysis.

### 2.4. Chemical analysis

All chemical analysis was performed in duplicate unless otherwise, specified. The WSB, experimental diets, and fecal samples were ground using a fixed blade laboratory mill (Retch, type ZM200, Haan, Germany) fitted with a 1.0-mm screen and stored in lidded glass jars in preparation. The ground WSB, experimental diets (after coating), and fecal samples were analyzed for DM, organic matter (OM), and ash according to the methods of Association of Official Analytical Chemists [(26); methods 934.01 and 942.05]. Crude protein (CP) content of the samples was analyzed using a nitrogen analyzer (FP928, LECO Corporation, Saint Joseph, MI) by the Dumas combustion method (AOAC 990.03). Acid hydrolyzed ether extract (AHEE) was determined by acid hydrolysis (AOAC 964.02). Gross energy (GE) was analyzed by a bomb calorimetry (Parr 6200 Calorimeter, Parr Instrument Company, Moline, IL). The titanium dioxide content in the samples was analyzed according to the colorimetric method described by Myers et al. (27). Total starch (TS) content of the samples was determined following the standard procedure from the Total Starch Assay Kit (K-TSTA-100A, Neogen, Lansing, MI). The TDF content of the samples was measured by following the standard procedure from the Total Dietary Fiber Assay Kit (K-TDFR-200A, Neogen, Lansing, MI). The WSB, rations, and dried extrudate samples were sent to a commercial analytical laboratory (Agricultural experiment station chemical laboratories, Columbia, MO) to determine phytate, urease activity, and trypsin inhibitor activity. Phytate was analyzed according to AOAC 986.11 method. Urease activity and trypsin inhibitor activity were analyzed according to AACC international approved methods of analysis (28, 29).

Ammonia concentration in the fresh fecal samples was analyzed according to the colorimetric method described by Chaney and Marbach (30). The fresh fecal samples kept frozen at −20°C for SCFA and BCFA analysis were thawed and diluted with deionized water and homogenized. The homogenized samples were centrifuged at 3,000 *g* for 20 min to separate the suspended solids. The 1 mL of the supernatant of the centrifuged samples was collected and 0.25 mL of 25% m-phosphoric acid was added to acidify the sample. The acidified samples were frozen at −20°C for at least 24 h to complete deproteinization. The acidified samples were thawed, centrifuged at 20,000 *g* for 15 min, and filtered through 0.2-μm PTFE filters with a syringe.

Fecal SCFA and BCFA contents were analyzed on a gas chromatography (GC) (31) equipped with flame ionization detector (FID) and a capillary column (BP-FATWAX UI, Agilent G3903-63008, 30 m × 0.25 mm × 0.25 μm, Agilent Technologies, Santa Clara, CA). Helium was used as a carrier gas with a flow rate of 40 cm/s, and the split ratio was 25:1 with injection volume of 0.5 μL. Hydrogen was used as the makeup gas with a flow rate of 25 mL/min. The detector and injector temperatures were set at 250°C, and the initial oven temperature was set to 80°C with a ramp rate of 10°C/min to 200°C for a total run time of 15 min. The peak area of chromatograms was determined using an integrative software (GC solution version 2.42.00, Shimadzu, Kyoto, Japan). The concentrations of SCFA (acetate, propionate, and butyrate) and BCFA (isobutyrate, isovalerate, and valerate) in the supernatant of the fecal samples were quantified by comparing the sample peak area to a standard with 10 mM of each volatile free acid (Volatile Free Acid Mix, Sigma-Aldrich, St. Louis, MO) and correcting for the fecal DM content.

### 2.5. Digestibility calculation

Two methods were utilized to estimate apparent total tract nutrient digestibility. The TFC method requires the collection of all feces excreted by the experimental animals. The marker method uses an indigestible dietary marker such as Cr_2_O_3_ or TiO_2_ (32). In the current study, apparent total tract digestibility (ATTD) of DM, OM, CP, CF, GE, and TDF was calculated according to the TFC (23) and marker methods (33):

(1) TFC method:


$$\begin{array}{l} Nutrient\;Digestibility,\;\%\\ =\frac{\left(nutrient\;consumed\;\left(g/d\right)\;-\;nutrient\;excreted\;\left(g/d\right)\right)}{\left(nutrient\;consumed\;\left(g/d\right)\right)}\;\times 100 \end{array}$$

(2) Marker method:


$$\begin{array}{l} Nutrient\;Digestibility,\;\%=\\ 1-\frac{\left(\%\;nutrient\;in\;feces\;\times \;\%\;TiO2\;in\;food\right)}{\left(\%\;nutrient\;in\;food\;\times \;\%\;TiO2\;in\;feces\right)\;}\times \;100 \end{array}$$

### 2.6. Palatability assessment

Palatability testing was conducted at a commercial research kennel (Summit Ridge Farms; Susquehanna, PA, USA) with a two-bowl test by beagle dogs (*n* = 20) for 2 days with each WSB containing diets compared to the control. Twenty male and female Beagles identified by ear tattoo and cage number were presented the test diets on an individual basis. Two stainless steel bowls, each containing ~400 g of diet, were offered once daily for 2 days. Bowl placement was reversed daily and both bowls were presented for 30 min. If one diet was completely consumed prior to the end of the 30 min, both bowls were removed. The total quantity of the food consumption and first choice (FC) preference were recorded for each dog. Individual intake ratios (IR) were calculated by dividing the intake of each diet into the total intake for both test diets. Preference was achieved by reviewing the average intake ratios for each dog in the test and scoring one point for the diet with an intake ratio ≥0.6667.

The kennel facility is registered with the USDA No. 23-R-0126 under the Animal Welfare Act. The kennel had a 12 h:12 h (light:dark) for each 24-h cycle and the temperature was controlled within targeted conditions range from 10 to 29.4°C in accordance with the Animal Welfare Act. Cages and food bowls were cleaned daily and sanitized in accordance with the Animal Welfare Act.

### 2.7. Statistical analysis

The nutrient ATTD, food intake, fecal output, fecal moisture, fecal score, defecation frequency, fresh fecal pH, SCFA, BCFA, and ammonia contents from the fresh fecal samples were analyzed using the GLIMMIX procedure of SAS (version 9.4, SAS Institute, Inc., Cary, NC). The treatment and period were the fixed main effects and dog (square) and square were random effect. Least square means of nutrient ATTD, fecal parameters, and fermentation parameters were analyzed with a single degree of freedom orthogonal contrasts. The *P*-values were reported for “control vs. treatments,” linear, quadratic, and cubic effects of nutrient ATTD and fecal parameters by dogs fed each treatment. The results were considered significant at *P* < 0.05 and trends were considered at 0.05 ≤ *P* < 0.10.

FC was analyzed by a Chi-square probability, and the consumption of each diet (control vs. treatment) was compared by a Wilcoxon signed rank test and a two-way analysis of variance (ANOVA). The results were considered significant at *P* < 0.05.

## 3. Results

### 3.1. Feed types and nutrient composition

The WSB used for this study (Table 2) contained 7.2% moisture. On DM basis, WSB contained CP (38.5%), CF (20.9%), TDF (19.2%), ash 5.1%, low total starch (1.2%), and GE (5,574.3 Cal/g), and the anti-nutritional factors for the WSB on as-is basis were Trypsin inhibitor (>16,000 TIU/g), urease activity (2.2 net pH increase), and phytic acid (1.2%). The WSB0 diet was within normal production parameters. The CP and OM (DM basis) were similar among diets (29.8 ± 0.65 and 93.8 ± 0.61%). Total starch content and GE tended to decrease as WSB was added to the diets but CF and TDF content tended to increase.

**Table 2:** Nutritional composition of raw WSB.

**Item**	**WSB**
Moisture, %	7.2
- Dry matter basis-	
Ash, %	5.1
Crude protein, %	38.5
Crude fat, %	20.9
Total starch, %	1.2
Total dietary fiber, %	19.2
Gross energy, kcal/kg	5,574
**Anti-nutritional factors (as-is)**	
Trypsin inhibitor, ^a^TIU/g	16,648
Urease, net pH increase	2.17
Phytic acid, ^b^W/W%	1.15

a TIU/g, Trypsin inhibitor unit per gram.

b W/W%, grams per 100 grams of sample.

### 3.2. Apparent total tract digestibility

All 12 dogs remained healthy throughout the study as confirmed by veterinary staffs at the Large Animal Research Center at Kansas State University (Manhattan, KS, USA). Dogs were fed with a certain amount of food to maintain body weight without any abnormal gastro-intestinal symptoms. This was confirmed; wherein, the BW of dogs on day 0 was 12.28 ± 1.51 kg and at day 56 was 12.82 ± 1.71 kg.

The apparent total tract digestibility of diets containing the WSB were evaluated by both, total fecal collection (TFC in Table 3) and by use of an indirect marker (titanium dioxide in Table 4). The variation (SEM) for the indirect marker method was smaller than TFC and, both data sets resulted in a linear decrease (*P* < 0.05) for ATTD of DM, OM, CF, and GE for dogs fed increasing levels of WSB diets. There was no linear decrease (*P* < 0.05) for ATTP of CP for the dogs in TFC method. There was no difference in TDF ATTD among treatments for TFC, while the TDF ATTD in WSB treatments were lower (*P* < 0.05) than the WSB0 when using indirect market method.

**Table 3:** Least square means and contrasts [WSB0 vs. WSB10–30 (*T*); linear (*L*); quadratic (*Q*)] for nutrient ATTD calculated using total fecal collection method (TFC) by dogs fed diets with increasing levels (0, 10, 20, and 30%) of WSB.

**Parameter**	**WSB0**	**WSB10**	**WSB20**	**WSB30**	**SEM**	**WSB0 v.s. *T***	***L***	***Q***
Dry matter, %	82.50	81.92	80.08	78.62	1.355	0.0246	0.0011	0.6037
- Dry matter basis -								
Organic matter, %	85.91	85.11	83.10	81.56	1.173	0.0028	< 0.0001	0.6052
Crude protein, %	86.29	86.01	85.36	84.92	1.075	0.3544	0.1891	0.9204
Crude fat, %	92.10	91.85	90.58	89.98	0.540	0.0040	< 0.0001	0.6358
Total dietary fiber, %	30.09	33.74	29.48	38.61	5.297	0.2722	0.1208	0.3652
Gross energy, %	86.40	85.61	83.94	82.30	1.0967	0.0061	0.0001	0.5577

**Table 4:** Least square means and contrasts [WSB0 vs. WSB10–30 (*T*); linear (*L*); quadratic (*Q*)] for nutrient ATTD calculated using an indirect marker method by dogs fed diets with increasing levels (0, 10, 20, and 30%) of WSB.

**Parameter**	**WSB0**	**WSB10**	**WSB20**	**WSB30**	**SEM**	**WSB0 v.s. *T***	***L***	***Q***
Dry matter, %	85.04	82.44	79.23	79.92	0.424	< 0.0001	< 0.0001	< 0.0001
**- Dry matter basis -**								
Organic matter, %	87.97	85.57	82.40	82.76	0.385	< 0.0001	< 0.0001	< 0.0001
Crude protein, %	88.23	86.52	84.67	85.91	0.430	< 0.0001	< 0.0001	< 0.0001
Crude fat, %	93.19	92.03	90.18	90.53	0.231	< 0.0001	< 0.0001	0.0011
Total dietary fiber, %	40.22	36.16	26.30	42.71	1.953	0.0003	0.6332	< 0.0001
Gross energy, %	88.37	86.05	83.27	83.44	0.387	< 0.0001	< 0.0001	< 0.0001

### 3.3. Hind-gut fermentation

There was a linear decrease (*P* < 0.05) in fresh fecal pH for dogs fed the WSB diets as WSB content increased (Table 5). The fecal NH_3_ concentration for dogs fed WSB containing diets tended to be lower (*P* = 0.054) than those for dogs fed the WSB0. The dog fecal acetic acid, propionic acid, total SCFA, and total fatty acids increased linearly (*P* < 0.05) as WSB levels increased. On the other hand, isobutyric acid, isovaleric acid, and the total BCFA content decreased linearly (*P* < 0.05) as diet WSB inclusion level increased in the diet. For these animals, the relative proportions of propionic acid and total SCFA increased linearly (*P* < 0.05) and those of butyric acid, isobutyric acid, isovaleric acid, and total BCFA decreased linearly (*P* < 0.05) as the WSB increased in the dog diets (Table 6).

**Table 5:** Least square means and contrasts [WSB0 vs. WSB10–30 (*T*); linear (*L*); quadratic (*Q*)] for fecal pH, short-chain fatty acid (SCFA), branched-chain fatty acid (BCFA), and total fatty acids (SCFA + BCFA) production from the fresh fecal sample collected from the dogs fed diets with increasing levels (0, 10, 20, and 30%) of WSB expressed in a μmol/g of feces in dry matter basis.

**Parameter, μmol/g of feces in dry matter basis**	**WSB0**	**WSB10**	**WSB20**	**WSB30**	**SEM**	**WSB0 v.s. *T***	***L***	***Q***
Fresh fecal pH	5.35	5.27	5.11	5.13	0.074	0.0337	0.01026	0.5063
Fecal NH_3_	92.45	77.10	75.58	75.84	7.326	0.0537	0.1123	0.2751
Acetic acid	173.03	211.97	218.77	203.37	9.925	0.0001	0.0071	0.0012
Propionic acid	67.85	99.87	107.86	105.14	6.229	< 0.0001	< 0.0001	0.0053
Butyric acid	34.34	37.35	32.76	22.22	7.492	0.6190	0.1465	0.2788
Isobutyric acid	2.29	1.74	1.53	1.29	0.262	0.0033	0.0017	0.4609
Isovaleric acid	4.68	3.16	2.49	2.23	0.470	< 0.0001	< 0.0001	0.1028
Valeric acid	0.79	1.01	1.01	1.11	0.1307	0.0981	0.1109	0.6318
SCFA^a^	275.23	349.20	359.40	330.72	17.835	0.0001	0.0087	0.0010
BCFA^b^	7.76	5.92	5.02	4.63	0.744	0.0011	0.0008	0.2508
TOTAL^c^	282.99	355.11	364.42	335.35	18.100	0.0003	0.0154	0.0015

a SCFA, short-chain fatty acids; sum of acetic acid, propionic acid, and butyric acid.

b BCFA, branched-chain fatty acids; sum of isobutyric acid, isovaleric acid, and valeric acid.

c TOTAL, total short-chain and branched-chain fatty acids; sum of SCFA and BCFA.

**Table 6:** Least square means and contrasts [WSB0 vs. WSB10–30 (*T*); linear (*L*); quadratic (*Q*)] for short-chain fatty acid (SCFA) and branched-chain fatty acid (BCFA) production from the fresh fecal sample collected from the dogs fed diets with increasing levels (0, 10, 20, and 30%) of WSB expressed as a percentage of total fatty acids.

**Parameter, %**	**WSB0**	**WSB10**	**WSB20**	**WSB30**	**SEM**	**WSB0 v.s. *T***	***L***	***Q***
Acetic acid	61.83	60.66	60.77	60.96	1.964	0.6260	0.7593	0.7117
Propionic acid	23.65	28.18	29.40	31.41	1.420	0.0004	0.0002	0.3433
Butyric acid	11.82	9.51	8.41	6.28	1.701	0.0183	0.0048	0.9462
Isobutyric acid	0.79	0.48	0.44	0.38	0.084	< 0.0001	< 0.0001	0.0354
Isovaleric acid	1.63	0.87	0.71	0.65	0.148	< 0.0001	< 0.0001	0.0015
Valeric, acid	0.27	0.29	0.28	0.33	0.040	0.5417	0.3370	0.6347
SCFA^a^	97.31	98.35	98.57	98.64	0.233	< 0.0001	< 0.0001	0.0070
BCFA^b^	2.69	1.65	1.43	1.36	0.233	< 0.0001	< 0.0001	0.0070

a SCFA, short-chain fatty acids; sum of acetic acid, propionic acid, and butyric acid.

b BCFA, branched-chain fatty acids; sum of isobutyric acid, isovaleric acid, and valeric acid.

### 3.4. Stool quality

There was no difference among the treatments in feed intake (Table 7). Generally, all foods were well-received and consumed by the dogs throughout the study, but the minor amounts of orts were measured and subtracted to calculate the food intake. If food was not readily consumed by dogs within 30 min, warm water was added in order to encourage food intake.

**Table 7:** Least square means and contrasts [WSB0 vs. WSB10–30 (*T*); linear (*L*); quadratic (*Q*)] for food intake, fecal output, fecal score, and defecation frequency of dogs fed diets containing increasing levels of WSB.

**Parameter**	**WSB0**	**WSB10**	**WSB20**	**WSB30**	**SEM**	**WSB0 v.s. *T***	***L***	***Q***
Intake (DM), g/d	163.95	174.61	171.62	163.30	10.385	0.4647	0.8732	0.1784
Fecal moisture, %	66.41	68.90	70.07	71.01	0.857	< 0.0001	< 0.0001	0.2292
Fecal output (As-is), g/d	82.02	97.11	111.23	119.11	5.369	< 0.0001	< 0.0001	0.4047
Fecal output (DM), g/d	27.30	30.06	32.86	34.35	1.367	0.0002	< 0.0001	0.5516
Fecal score^a^	3.94	3.93	3.91	3.87	0.069	0.3959	0.1969	0.7417
Defecation frequency, times/day	1.80	1.85	1.93	2.12	0.101	0.0989	0.0106	0.4378

a Subjective 1 to 5 scale with 1, runny; 2, soft; 3, firm and moist; 4, firm; 5, dry and hard.

The fecal moisture, wet fecal output, and dry fecal output were greater (*P* < 0.05) for dogs fed WSB containing diets relative to dogs fed the control diet. These variables increased in a linear fashion (*P* < 0.05) as more WSB were included in the diet. Despite this, the fecal scores were consistent among all treatments (*P* > 0.05) with scores average 3.9 ± 0.03. The defecation frequency of dogs did not differ between those fed the control diet and dogs fed the WSB containing diets (*P* > 0.05), but there was a linear increase (*P* < 0.05) in the defecation frequency as dogs were fed increasing WSB in experimental diets.

### 3.5. Canine palatability

In all cases the WSB containing diets were preferred by dogs (*P* < 0.05) relative to the control (WSB0) in FC assessment (Table 8). When comparing WSB10 to WSB0, 27 occasions out of 40 were chosen for WSB10 over WSB0 by dogs (*P* < 0.05). The 28 occasions out of 40 were chosen for WSB20 over WSB0 by dogs (*P* < 0.05). The 29 occasions out of 40 were chosen for WSB30 over WSB0 by dogs (*P* < 0.05). There was no difference for the food consumption and intake ratio (IR) between the WSB containing diets and the WSB0 control.

**Table 8:** Palatability assessment of diets containing WSB relative to the control (0% WSB) by dogs.

**Diet A vs. B**	**FC, n^a^**	**IR of diet A^b^**
WSB10 v.s. WSB0	27^*^	0.577
WSB20 v.s. WSB0	28^*^	0.615
WSB30 v.s. WSB0	29^*^	0.632

a FC (first choice) number of first visits to bowl with diet A can be obtained by 40-n.

b IR (intake ratio) of diet A = average of intake (g) of diet A/total intake (g) of diets A + B.

* P-value is < 0.05.

## 4. Discussion

### 4.1. Feed types and nutrient composition

The CP and CF (DM basis) of WSB used in this study (39 and 21%, respectively) were within the range reported in the literature [full-fat soya flour, 42 and 22%, (34); and raw soybean seed meal, 37 and 22.0%, (35); raw soybean 40 and 21.7%, (36)]. The experimental diets were formulated to be isonitrogenous and isocaloric, but the internal fat and TDF included in WSB gradually increased the CF and TDF content of the diets as the WSB inclusion level increased.

### 4.2. Apparent total tract digestibility

The dogs were healthy throughout the feeding trial. Their initial BW remained constant and there were no differences in feed intake among the treatments, which is consistent with other research evaluating soy in pet food (37). Furthermore, Menniti et al. (38) evaluated blood parameters of healthy, adult dogs fed SBM as a replacement of up to 30% of protein provided from chicken. In their study, all blood parameters remained within normal physiological ranges. Blood chemistry was not analyzed in the current study, which is a limitation and a potential future research opportunity.

The ATTD results from TFC method can be considered by some as the gold-standard presuming that all feces are collected, and there is no loss of feces due to coprophagy. Alvarenga et al. (32) pointed that the TFC method may lead to an overestimation of ATTD compared to the indirect marker method due to instances of loss of fecal samples by either daily pen sanitation or liquid diarrhea. However, the results from the current experiment would suggest that both TFC and indirect marker method were valid and led to the same interpretation based on statistical data analysis.

According to several studies that evaluated dietary SBM as a protein source in dog foods, CP ATTD would not be negatively affected (38–41). However, in current study, the inclusion of WSB into the diets decreased nutrient digestibility of DM, OM, CP, CF, TDF, and GE for dogs compared to the control, WSB0. The lower nutrient digestibility in WSB-containing diets in this study could be explained by two main effects derived from WSB: fiber content from the soybean hulls and the residual level of anti-nutritional factors from WSB. Soybean hulls contained more than 60% TDF (14) consisting of hemicellulose, cellulose, and pectin (42). Cellulose is composed of strands of glucose units which are linked by 1–4 β-bonds (43). The fiber structure might have limited the enzymatic hydrolysis of the substrate, affecting nutrient bioaccessibility and the extent of macronutrient digestion and absorption (44). On the other hand, Colonna et al. (45) noted that dietary fibers form gels in the gastrointestinal tract and limited enzymatic hydrolysis. Moreover, Burrows et al. (46) and Fahey et al. (47) found that the DM digestibility and the intestinal transit time in dogs decreased with the addition of fibers. They concluded that the decreased intestinal transit time contributed to the depression of DM digestibility.

From the work of Kim and Aldrich (6), the single extruder extrusion of the diets containing WSB did not eliminate the anti-nutritional factors of soybeans. Other soybean products like SBM were subjected to various processes, such as cleaning, dehulling, conditioning, flaking, boiling, or toasting, and oil extraction by either mechanical method or solvent extraction (48). The defatted soybean flakes, following oil extraction are typically subjected to processing conditions with different range of moisture, temperature and drying time to produce SBM (49). The WSB used in this study was raw full-fat soybean that was intact and contained high level of anti-nutritional factors prior to extrusion with the other ingredients. Felix et al. (50) evaluated different soy protein products and reported that WSB had the highest urease and trypsin inhibitor (TI) even after the diet extrusion. Among all anti-nutrients present in foods, TI are of great importance since they affect protein utilization and digestion (36). The TI interferes with protein digestibility by forming an irreversible trypsin enzyme-trypsin inhibitor complex that declines trypsin enzyme in the small intestine (51). The negative impact due to the presence of high TI concentration in WSB-containing diets on the CP digestibility occurred in this study. Fermentable carbohydrates, such as OS may impact nutrient availability by affecting transit time and forming complexes between fibrous compounds and other nutrients (52). Smiricky et al. (53) reported that inclusion of soy OS reduced nutrient digestibility in growing pigs, but the reduction was small.

The WSB contained ~21% fat (DM basis) within the seed. The internal fat derived from the WSB could have a lubricating effect during processing in the single-screw extruder. Soybean products that are high in fat content, such as soybean and micronized soybeans, reduced starch gelatinization in extruded dog foods (50). The addition of fat to the ration reduced shear inside the extruder, thereby reducing the specific mechanical energy and die temperature (6). Digestibility of starch could be affected by the degree of thermal processing since the gelatinization degree has implications on starch utilization in dogs. Less cooked starches contain a higher proportion of resistant starch and are less digestible in dogs (54). The decreased degree of cooking as we increase the WSB level in diets might have reduced the DM ATTD in this study. While these differences were significant statistically, the overall level of digestibility among the diets was high (e.g., average DM ATTD 82 ± 2.6%; CP ATTD, 86 ± 1.5%; CF ATTD, 91 ± 1.4%) compared to the previous research that evaluated ATTD of SBM containing diets (e.g., DM ATTD, 80 ± 3.5%; CP ATTD, 83 ± 2.6%; CF ATTD, 88 ± 9.5%) (55).

### 4.3. Hind-gut fermentation

In this study, fermentable carbohydrates derived from WSB might have decreased fresh fecal pH and ammonia concentration in dogs. Moreover, the acetate, propionate, and total SCFA concentrations increased while the total BCFA concentration decreased as the WSB inclusion level increased in the diet. Félix et al. (56) confirmed that the fermentation of the high non-digested carbohydrates lowered the fecal pH of the dogs fed diets containing SBM. Middelbos et al. (57) reported an increase in SCFA in beet pulp treatment compared with the control and cellulose treatments. According to Tortola et al. (41), the SBM intake increased the fecal concentrations of propionate, acetate, and total SCFA and reduced ammonia and fecal pH, which corresponds to the current study. They concluded that soybean OS were the fermented substrate by the gut microorganisms in dogs given that the diets were similar in dietary fiber content.

Soybean OS are potential prebiotics since they are rich in galactooligosaccharides, namely stachyose, raffinose, and verbascose (58). Other reported major sugar of soybeans is sucrose, with a lower quantity of monosaccharides (59). According to Grieshop et al. (60), the average stachyose, raffinose, verbascose, and sucrose contents on DM basis of 10 different soybeans was 3.8, 0.6, 0.2, and 4.8%, respectively. Similarly, Berk (61) reported that soybeans contained 4% stachyose, 1% raffinose, and 5% sucrose. The α-galactosidic bond between sucrose and galactose that occurs in the galactooligosaccharides cannot be hydrolyzed in the small intestinal tract due to the lack of α-1,6-galactosidase (62). Intact OS are fermented by the colon microorganisms that contain α-galactosidase (63) such that the non-digestible OS are indirect energy substrates and metabolic regulators (16). Besides the OS that is not captured from the TDF analysis, the carbohydrate in soybean consists of non-starch polysaccharides (NSP) (64). Kim et al. (11) reported that soybean hulls contained 71.5% of total NSP. Main non-cellulosic polysaccharides from soybean hulls were mannose and xylose (65).

Fermentation of dietary fibers including NSP and OS resulted in the production of SCFAs (mainly acetate, propionate, and butyrate), which reduced the pH of the intestinal lumen (66). The amounts, types, and the production rate of SCFA produced in the colon depended on the source of non-digestible carbohydrate substrate and the intestinal microflora (16). Acetate is partly taken-up by the liver and can be oxidized by muscle tissues throughout the body for energy in dogs (67). Propionate is quantitively removed from portal blood by the liver and either used as substrates for gluconeogenesis or involves in Krebs cycle at the level of succinyl coenzyme A (66). On the other hand, butyrate is extensively metabolized in the colon. Butyrate serves as the preferred energy substrate of colonocytes (68, 69). Undigested proteins are fermented and form fermentation metabolites such as BCFA, ammonia, phenolic and indolic compounds, biogenic amines, hydrogen sulfide, and nitric oxide (70). Nery et al. (71) reported that the feeding high protein diets led to greater fecal concentrations of ammonia, BCFA, and valerate. Ammonia, amines, and sulfide are known to be potentially harmful in animals (72) by shortening the colonocytes life span (73) and promoting tumor growth (74). Anaerobic bacteria in the colon assimilate ammonia to form microbial protein during carbohydrate fermentation, so the ammonia concentration in the large intestine depends on the balance between amino acid deamination and bacterial protein synthesis (74). Thus, an increase in SCFA and a decrease in pH, BCFA, and ammonia could be interpreted to positively affect intestinal health (25).

### 4.4. Stool quality

In previous research, fecal output and score data were highly related to the TDF and non-structural carbohydrate content of soy containing diets (75). The water-holding capacity of fiber is known to have a physiological effect on fecal bulking and shorten gut transit times, resulting in increased fecal weight and stool frequency (76). According to Bednar et al. (77), the soluble dietary fiber (SDF) fraction can increase the water-holding capacity of digesta resulting in greater fecal output. Muzilla et al. (78) also reported that heat significantly increased water absorption of soy hulls which is a component of WSB. Insoluble dietary fiber (IDF) contributed to fecal bulk and promoted laxation (14). Therefore, the linear increase in fecal output, fecal moisture, and defecation frequency for dogs fed increasing levels of WSB-containing diets might be attributed to the increasing TDF content in the diets. These results are consistent with the previous studies (38, 39, 79, 80).

### 4.5. Canine palatability

The two-bowl forced-choice evaluation is a common method for palatability evaluation in dogs (81). The IR was used to determine the preference by quantifying whether one or the other bowl was consumed in a greater proportion (82). In the two-bowl test, the animal is allowed to smell the food before the consumption, and then the first bite from either food is recorded as FC. Thus, the FC is related to the aromatic characteristics of the food.

In the current study, dogs favored WSB containing diets over the control diet for FC, which is an indicator of aroma, but this did not result in higher consumption. Dogs are known to prefer high-fat (83, 84) and less-fibrous foods (85). In the current study, WSB-containing diets had higher FC compared to WSB0. This was interesting since the amount of chicken fat applied to coat the diets were lower in WSB-containing diets than in WSB0 even though the CF content of WSB-containing diets was higher than WSB0 due to the internal fat derived from the WSB. This study found that dogs preferred higher fat containing diets from an aromatic perspective, with no preference between chicken fat and soybean oil. Similarly, Inal et al. (86) found that sunflower oil was preferred over poultry fat by dogs. In contrast, Félix et al. (79) reported that dogs preferred SBM-based diets over diets with poultry meal for total food consumption. It was suggested that the content of low molecular weight sugars in SBM may contribute to its greater preference by dogs (79). The WSB-containing diets had higher TDF than WSB0. According to Koppel et al. (85), dogs preferred control diets over diets containing higher dietary fiber (sugar cane or wheat bran fibers). The higher fat content of the WSB diets might have driven higher FC in dogs, but the higher TDF content of WSB diets limited the food consumption leading to no difference in IR. This would suggest that there were no palatability issues with the increasing levels of WSB in the diet and that the size, shape, density, and texture of the product noted in the processing work (6) was not deleterious to the product acceptability by the dogs.

## 5. Conclusions

In conclusion, incremental dietary level of WSB from 0 to 30% was not harmful or deleterious to dog stool quality and palatability in this experiment. In contrast, the higher inclusion of WSB decreased the nutrient ATTD in dogs, but all levels remained high. The WSB increased the hind-gut fermentation of the diets, which can be useful to make high fiber diets for geriatric dogs or less-active adult dogs for their gut health.

## Data availability statement

The original contributions presented in the study are included in the article/supplementary material, further inquiries can be directed to the corresponding author.

## Ethics statement

The animal study was reviewed and approved by Kansas State University Institutional Animal Care and Use Committee (IACUC).

## Author contributions

CA and HK formulated the diets and designed the study. HK collected samples, performed statistical analysis, and primary writing. HK and SL performed sample analysis. YZ and CA provided editing and interpretation. All authors contributed to the article and approved the submitted version.
